# Pre-stimulus pupil dilation and the preparatory control of attention

**DOI:** 10.1371/journal.pone.0188787

**Published:** 2017-12-08

**Authors:** Jessica L. Irons, Minjeong Jeon, Andrew B. Leber

**Affiliations:** 1 Department of Psychology, The Ohio State University, Columbus, United States of America; 2 Graduate School of Education & Information Studies, University of California, Los Angeles, Los Angeles, United States of America; University of Verona, ITALY

## Abstract

Task preparation involves multiple component processes, including a general evaluative process that signals the need for adjustments in control, and the engagement of task-specific control settings. Here we examined the dynamics of these different mechanisms in preparing the attentional control system for visual search. We explored preparatory activity using pupil dilation, a well-established measure of task demands and effortful processing. In an initial exploratory experiment, participants were cued at the start of each trial to search for either a salient color singleton target (an easy search task) or a low-salience shape singleton target (a difficult search task). Pupil dilation was measured during the preparation period from cue onset to search display onset. Mean dilation was larger in preparation for the difficult shape target than the easy color target. In two additional experiments, we sought to vary effects of evaluative processing and task-specific preparation separately. Experiment 2 showed that when the color and shape search tasks were matched for difficulty, the shape target no longer evoked larger dilations, and the pattern of results was in fact reversed. In Experiment 3, we manipulated difficulty within a single feature dimension, and found that the difficult search task evoked larger dilations. These results suggest that pupil dilation reflects expectations of difficulty in preparation for a search task, consistent with the activity of an evaluative mechanism. We did not find consistent evidence for relationship between pupil dilation and search performance (accuracy and response timing), suggesting that pupil dilation during search preparation may not be strongly linked to ongoing task-specific preparation.

## Introduction

Every day we engage in a variety of complex, cognitively demanding tasks. For each new task, the attentional control system must be reconfigured to ensure that only currently relevant information is prioritized. For example, when preparing to pull out from a car park on the street, attention must be configured to focus on approaching cars or pedestrians crossing the street, and ignore competing visual input from stores and cafes nearby. This act of preparing the attentional control system for a new task can have a significant impact on how effectively the task is performed.

Preparing for a new task involves multiple distinct and interconnecting processes. A prominent view in cognitive control research distinguishes between two stages: an evaluative stage followed by task-specific preparation. The evaluative mechanism monitors ongoing task performance and assesses the need for adjustments in control [1–4]. This mechanism has been linked to activation in the anterior cingulate cortex (ACC), and is sensitive to situations involving a high degree of conflict (e.g. incongruent Stroop trials [5]) and those in which errors are expected [6]. The results from the evaluative process determine whether, and to what extent, task-specific preparation should be engaged. This second stage involves the activation of task sets, cognitive settings tailored to the operations that must be performed in the upcoming task [7–9]. Neural measures of task set activation have been shown to be directly associated with response time on the task [10, 11]. For many attention tasks, task-specific preparation involves engaging attentional control settings, which specify the features or properties of task-relevant stimuli [12]. These attentional control mechanisms are subserved by prefrontal and parietal brain regions [13–17]. The preparatory activation of attentional control settings, as evidenced by a pre-stimulus increase in neural activity at regions of the visual cortex sensitive to the target feature dimension, predict subsequent search accuracy and response time [18–21].

In the current study, we sought to explore components of attentional task preparation using pupillometry. Pupillometry has been widely used to make inferences about task-evoked processing demands. Engaging in cognitive processing evokes a phasic dilation of the pupils, a response that has been attributed to inhibition of the parasympathetic autonomic system under the control of the locus coeruleus-norepinephrine system [22–24].

Task-evoked dilations occur in response to a wide variety of cognitive operations, including target engagement and identification (e.g., [25]), conflict processing (e.g., [26]), memory encoding and retrieval (e.g., [27, 28]), and motor preparation (e.g., [29, 30]). Importantly, the magnitude of the dilation is typically correlated with the cognitive demands of the task. For example, early demonstrations by Hess and Polt [31], and well as Kahneman and colleagues [32, 33] showed larger dilations for tasks with higher cognitive load (e.g. maintaining seven digits in working memory) than those with a smaller load (e.g. maintaining three digits in working memory). Kahneman suggested that pupil dilations reflect the active exertion of mental effort brought to bear on a task.

In relation to visual attention, pupil dilation has been recruited to help elucidate mechanisms underlying visual search. For example, studies by Porter and colleagues [34–36] suggest a link between pupil dilation and visual search efficiency. Engaging in a difficult, inefficient feature search (e.g. a target amongst heterogenous distractors) was associated with larger dilations than engaging in an easy, efficient feature search (e.g. a target amongst homogenous distractors) [36]. Changing the properties of the target (conjunction versus feature target) did not affect pupil dilation, provided that efficiency was matched ([35], but see [34] for evidence that older adults with Alzheimer’s disease do show larger dilations for conjunction over feature search).

Pupil dilation has also recently been shown to be sensitive to the moment-by-moment demands on attentional control. In a visual search task, Mathot and colleagues [37] found that greater dilations predicted eye movements to display locations that were low in salience compared to regions high in salience. Given that greater attentional control is required to override salience, these results are consistent with the notion that pupil dilations provide an online measure of attentional control. Further, the predictive nature of the relationship between dilation and eye movements suggests that dilations may have been tapping into task-specific preparatory processing. These results motivate several further questions. First, the observed relationship between dilation and behavior was correlational, not experimentally manipulated. When task conditions are manipulated experimentally (e.g., via external cue designating upcoming task characteristics), will dilation still track with task parameters? Second, pupil dilations were measured during ongoing task performance, when the to-be-fixated stimulus was visually available. This leaves the possibility that some aspect of the available scene could have interacted with the pupil response, although the experimenters went to appreciable lengths to control for this. Would pupil dilation predict upcoming task parameters in the absence of exposure to the visual scene?

One approach to address these follow up questions is to measure pre-stimulus dilation in anticipation of performing a task. Previous studies have shown that pupil dynamics are sensitive to preparatory processing. For example, Wang, and colleagues [38] found that pupil dilation is larger in anticipation of performing an anti-saccade (away from a probe) than making a pro-saccade (towards a probe). Importantly, the dilation for both pro- and anti-saccades was negatively correlated with the latency of the saccade, such that greater dilation predicted faster responses. Wang et al. [38] interpreted their result as showing preparatory engagement of the task set, which, for anti-saccades, included the activation of top-down inhibition. Similarly, Boehler and colleagues [39] measured dilation while cueing participants to perform an easy or difficult target discrimination task. Pre-stimulus pupil dilation was larger in response to difficult than easy cues, which was attributed to differences in task demand. However, because the easy and difficult tasks were blocked, it is not clear whether pupil dilation was modulated by expectations of the upcoming task demands or as a reaction to the previously experienced task.

Here we report a new study in which we measured pre-stimulus pupil dilation to investigate attentional task preparation. Specifically, we were interested in assessing the component processes of evaluating attentional demands and engaging task-specific control mechanisms. We analyzed pupil dilation during the lead-up to performing a visual search, allowing us to isolate preparatory processing from ongoing visual stimulus processing. We experimentally manipulated the attentional control demands of the search task by varying the salience of the target, on the assumption that more control would be required to search for lower salience targets. We addressed two main questions: 1) Does pupil dilation reflect evaluative processing, responding to the anticipated attentional demands of the upcoming search task? and 2) Does pupil dilation reflect task-specific processing, in which the magnitude of pupil dilation predicts search accuracy and/or response time?

To preview the results of three experiments, we found that mean pupil dilation was sensitive to expectations of difficulty, but trial-by-trial dilation did not reliably predict task performance. We thus will conclude that pre-stimulus pupil appears linked to evaluative processing, but we make no strong claims about how it relates to task-specific processing and leave this latter issue open to further investigation.

## Experiment 1

**Fig 1:**
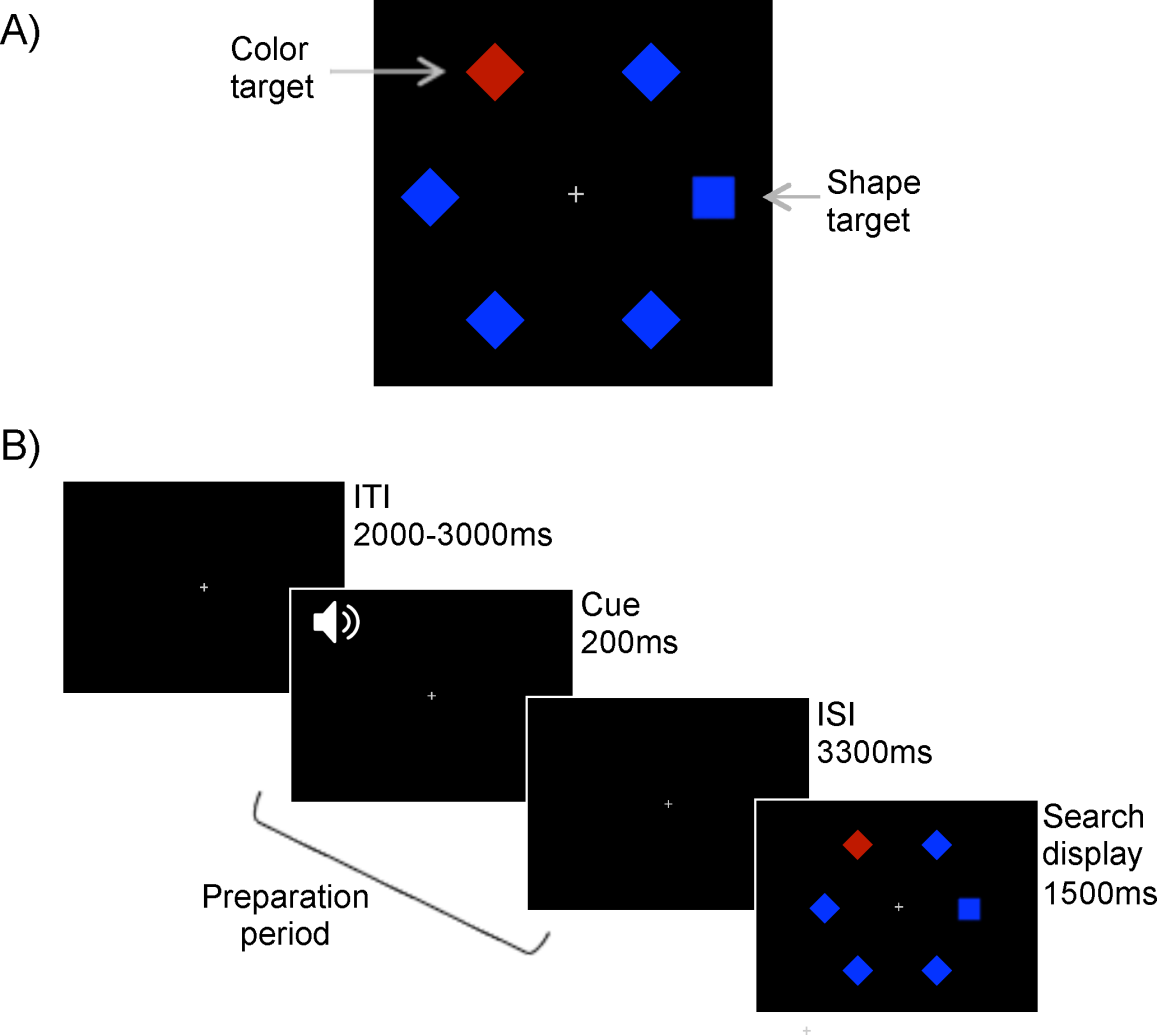
**Experiment 1 search display (A) and trial sequence (B).** Targets were either a color singleton or shape singleton, and were cued by a high or low pitched tone at the start of each trial. Participants responded by making a saccade to the target.

In this initial experiment, participants searched for one of two possible targets in a search display: a color singleton and a shape singleton (Fig 1A). Typically, color singletons are more salient than shape singletons [40], and we accentuated this by purposely choosing a very distinct color singleton (e.g., red amongst blue distractors) and a non-distinct shape singleton that shared similar properties with the distractors (square amongst diamonds). An auditory cue at the start of each trial indicated the target to be detected. This was followed by a preparation period of 3.5 seconds before the presentation of the search display (Fig 1B), and pupil dilation was recorded across the period.

We measured the contributions of the two preparatory processes in different ways. We predicted that evaluative processing would emerge as significantly larger pupil dilation in anticipation of the more difficult shape target than the easier color target. Here, the effect would be driven by the expectations of the observer regarding the difficulty of the upcoming task and the corresponding need for control. Task-specific preparation, although linked to evaluative processing [1], must be measured using more stringent criteria. Specifically, greater task preparation logically should lead to better behavioral outcomes, as reflected on a trial-by-trial basis (e.g., [10, 14, 41]). That is, task-specific preparation should manifest as a relationship between the pupil signal and behavior. Specifically, we predicted that the magnitude of the pupil dilation would predict subsequent search accuracy and/or RT.

### Method

#### Participants

Participants were 16 undergraduate students from The Ohio State University (7 female, 9 male; age range 18–20, *M* = 18.87), and they received either psychology course credit or $10 for participating. We chose this sample size on the expectation that it would yield useful data for initial analysis and motivate follow-up experiments; we did not conduct any power analysis. In all three experiments, participants reported normal or corrected-to-normal vision and normal color vision and gave informed written consent to participate. The methods for this experiment, as well as for the two subsequent experiments, were approved by The Ohio State University institutional review board and adhere to the Declaration of Helsinki.

#### Stimuli and equipment

All stimuli were presented against a black background, and a small grey fixation cross remained in the center of the screen throughout the task. The cues were a high pitch (700Hz) and a low pitch (500 Hz) auditory tone. The search display consisted of six items, each approximately 1.4° x 1.4°, arranged in a ring with a radius of 7.2° around fixation. The items comprised a color singleton target, a shape singleton target and four distractors. The color singleton target was colored either red (RGB: 175, 0, 0; CIE Lab: 45.07, 70.65, 59.28) or blue (RGB: 0 0 255; CIE Lab: 32.30, 79.19, -107.86), whereas the shape singleton and the distractors were all presented in the non-target color (i.e., if the color target was red, the distractors were blue). The shape singleton target was always a square and all other items were diamonds (i.e., a square rotated 45°). To approximately match the perceived luminance of red and blue, one of the experimenters self-ran the flicker photometry method [42], prior to data collection. This process involved presenting red and blue patches in the center of the screen, alternating at a rate of 85Hz. The blue patch was used as the reference, and the intensity of the red hue (in RGB values) was adjusted until the two fields had appeared to fuse and flickering was at a minimum, at which point the two colors are considered of approximately equal subjective brightness. This was repeated 10 times, and the average of the resulting red hues was used for the experiment.

The experiment took place in a dimly lit and sound attenuated individual testing room. Pupil area was measured using an Eyelink 1000 desk-mounted eye tracker (SR Research, Mississauga, ON, Canada), tracking the left eye. Stimuli were presented on a 20-inch ViewSonic CRT monitor with a refresh rate of 85Hz, using Psychophysics Toolbox extensions for Matlab [43, 44] with Matlab (Mathworks, Natick, MA). Viewing distance was held constant at 60cm from the computer monitor, enforced with a chin rest.

#### Procedure

Participants were instructed that they should search for either the color or the shape target on each trial, depending on the auditory cue presented at the start of the trial. Both targets were present in every display, making it necessary to use the cue to determine the correct target. For half of the participants, the high-pitch cue indicated that they should search for the color target and the low-pitch cue indicated that they should search for the shape target. For the remaining participants, the cue-target pairings were reversed. Whether the color target was red amongst blue or blue amongst red was also counterbalanced across participants.

Participants were asked to maintain fixation in the center of the display until the search display was presented. Each trial began with the fixation display for a variable interval of 2000, 2500 or 3000ms (each duration presented equally often), followed by the cue for 200ms. The fixation display remained on the screen during the cue and for a further 3300ms, giving a preparation period of 3500ms from cue onset to search display onset. The search display was presented for 1500ms. Participants responded by fixating the target as quickly and accurately as possibly. A response was logged when the eyes moved more than 6° from fixation (within about 1.2° of the ring formed by the centers of the search items), and was assigned to the closest item. If a non-target item was fixated before the target, the trial was marked as incorrect. Feedback on error trials was given via a short beep.

Participants completed 15 practice trials, followed by eight blocks of 30 experimental trials (240 trials total). The eye tracker was recalibrated at the start of each block. Each block comprised 15 color target and 15 shape target trials. The trials were presented in random order, with the restriction that the number of trials requiring a repetition (i.e., the target on trial N is the same as the target on trial N-1) and those requiring a switch (the target on trial N is different to the target on trial N-1) were as equivalent as possible. The spatial locations of the two targets were completely randomized.

#### Analysis of pupil data

Pupil area was recorded every 2ms, from 100ms before cue onset to the end of the preparation period, in arbitrary units provided as default by the Eyelink software. The standard Eyelink blink detection algorithm was used to establish a start and end point for each blink. As the impact of blinks has been shown to extend beyond the period defined by Eyelink, an extra 60ms before the start of the blink and 150ms after the end of the blink were added to the blink period [45, 46]. Pupil area during blinks was replaced using linear interpolation. Any participant missing more than 40% of their pupil data (e.g., due to blinks) was excluded from the sample (following [47]).

Pupil area measurements were downsampled to 10Hz by taking the median pupil area for every 100ms bin. This resulted in 36 binned values per trial. The first bin, covering the 100ms period before the cue, was designated as the trial baseline. The remaining 35 bins covered the preparation period. To control for possible individual differences in pupil size and sensitivity, all binned values across the entire experiment were z-scored separately for each participant. Dilations were calculated by subtracting the trial baseline from each binned value in the trial. Finally, a mean dilation was calculated for each trial by averaging across all 35 dilation values.

We conducted two main analyses of the pupil data. First, we assessed the main effect of target type on dilation. To determine whether the expected target type yielded differences in preparatory pupil dilation, we conducted within-subjects t-tests on color vs. shape target trials. Second, we analyzed the relationship between dilation and task performance. To assess whether pupil dilation predicted search performance on a trial-by-trial basis, we used a linear mixed effects model fit with R package lme4 (version 4_1.1–12 [48]). We specified a model where pupil dilation was the main fixed effect of interest, and accuracy and RT were the dependent variables. Because the distribution of RTs tends to be positively skewed, we used logarithmically transformed RT values. We also included fixed effects for any control variables that we predicted might influence the relationship between performance and dilation, including target type (color or shape), target type on the previous trial, accuracy (for RT analyses only), accuracy on the previous trial, baseline pupil area (z-scored), and inter-trial interval. To control for variation across trials and across individuals, we added random intercepts for trials and participants. We tested significance by comparing the full model with a reduced model (including all fixed and random effects except pupil dilation) and used a likelihood ratio test to determine whether adding mean dilation to the model significantly improved the goodness of fit. Additionally, we examined whether the relationship between dilation and task performance (accuracy and RT) interacted with target type, by adding a dilation x target type term into the model and comparing this new version to the original model.

### Results and discussion

#### Behavior

Analysis of the behavioral data confirmed that performance was influenced by target type. Accuracy was significantly lower on low-salience shape target trials (*M* = 69.94%) compared with high-salience color target trials (*M* = 81.19%; *t*(15) = 4.23, *p* < .001, *d* = 1.06). Saccadic response time analyses were conducted on accurate trials only, excluding trials with RTs < 100ms (reflecting pre-emptive saccades and comprising 4.82% of trials) or more than three standard deviations above the mean for color or shape trials (6.22% of trials). As expected, saccadic response times were slower for shape than color trials (*Ms* = 426ms and 590ms respectively; *t*(15) = 6.45, *p* < .001, *d* = 2.29).

#### Main effect of target type on dilation

**Fig 2:**
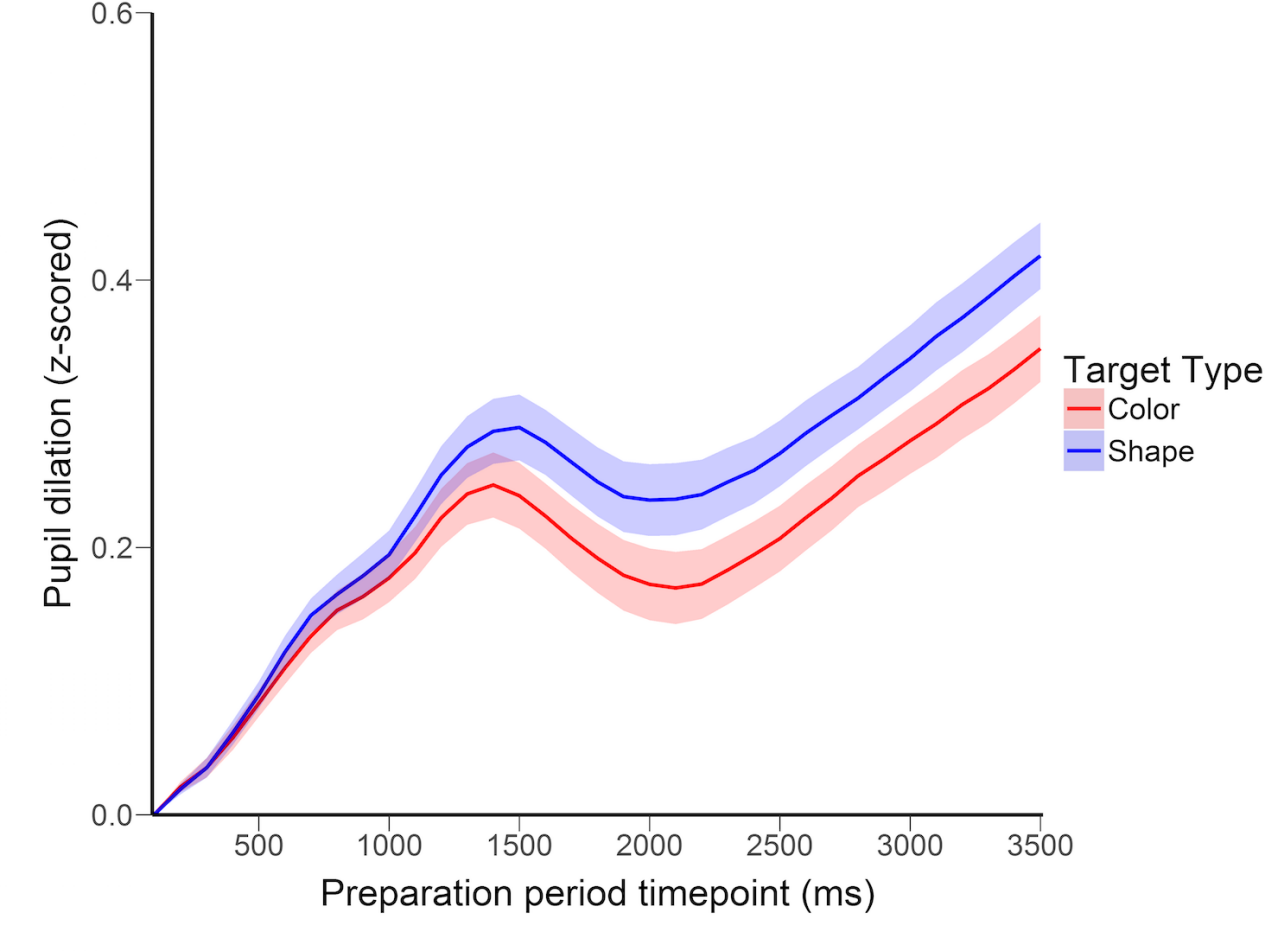
Pupil dilation across the preparation period in Experiment 1. Pupil dilation was calculated using z-scored pupil area values. Error bands depict standard error of the mean color vs shape difference scores.

Analysis of pupil dilation revealed a significant effect of target type. Mean pupil dilation was significantly larger when preparing for the more difficult shape target compared with the easier color target, *t*(15) = 2.37, *p* = .032, *d* = .60. As shown in Fig 2, dilations across color and shape trials began to diverge as early as 500ms after cue onset, reaching maximal separation at approximately 2000ms and maintaining this separation until the presentation of the search display.

#### Relationship between dilation and task performance

**Fig 3:**
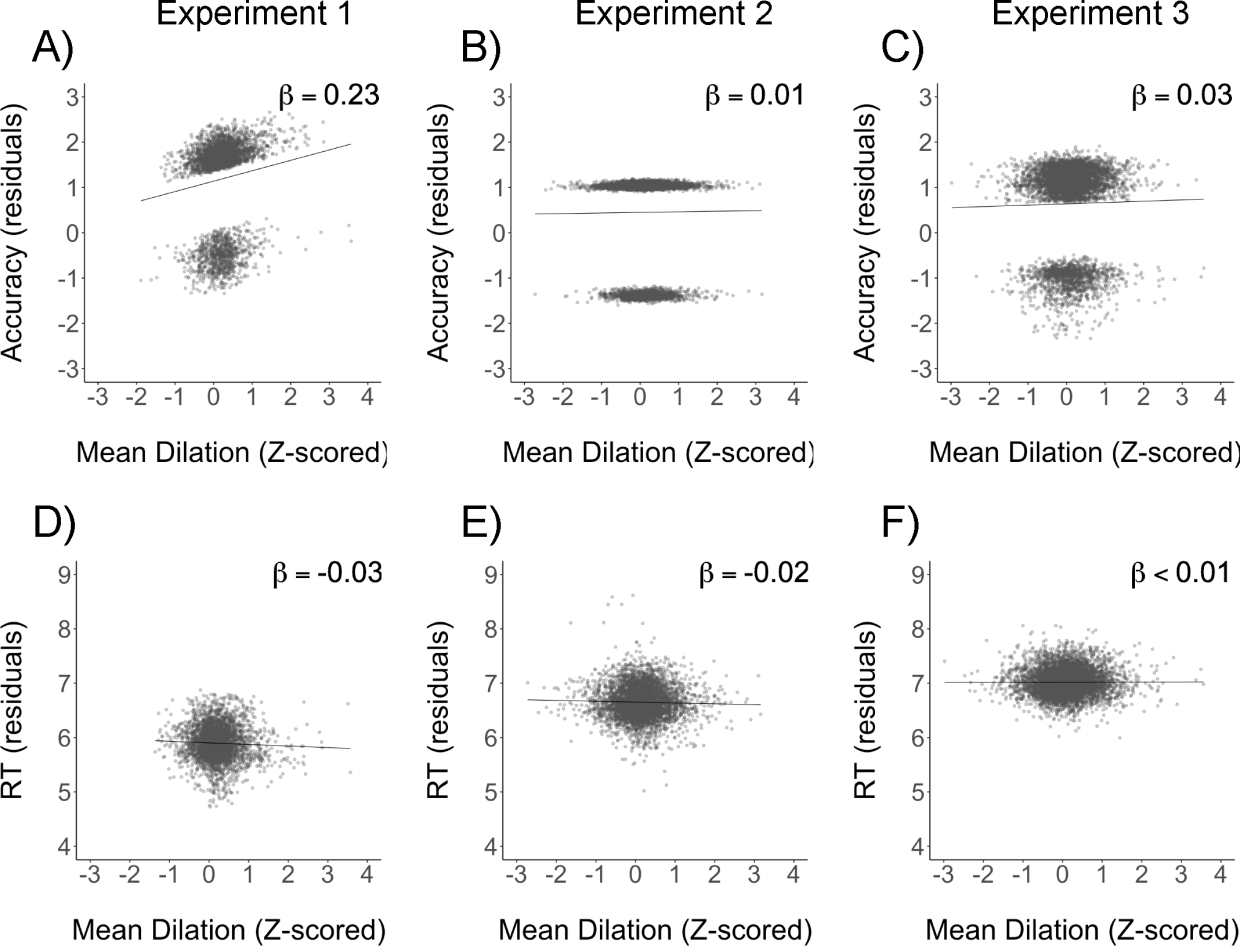
Partial regression plots of linear mixed effects results from Experiments 1–3. (A)–(C) show accuracy residuals after controlling for all other fixed and random effects plotted against mean dilation for Experiment 1 (A), Experiment 2 (B) and Experiment 3 (C). Accuracy was coded as 0 for incorrect and 1 for correct, and consequently correct and incorrect trial residuals form separate clusters of dots in the scatterplots. (D)–(F) show RT residuals after controlling for all other fixed and random effects plotted against mean dilation for Experiment 1 (D), Experiment 2 (E) and Experiment 3 (F).

We next examined whether pupil dilation predicted trial-by-trial search performance (accuracy and log-transformed RT) using a linear mixed effects model (see Table 1 for a summary of the model and results from all three experiments). First, we found that pupil dilation was a significant predictor of accuracy, with larger dilations associated with greater likelihood of making a correct response (β = .23, *SE* = .09). Removing pupil dilation from the model significantly decreased the goodness of fit (likelihood ratio test χ^2^ (1) = 5.76, *p* = .016). In Fig 3A, accuracy residuals (i.e., after controlling for all other fixed and random effects) are plotted against mean dilation with each dot representing an individual trial, demonstrating the predictive effect of mean dilation. Note that because accuracy was a binary variable (where 0 = incorrect and 1 = correct), correct and incorrect trials form separate clusters of dots in the scatterplot. Pupil dilation did not interact with target type (*p* = .70), suggesting that the relationship between pupil dilation and accuracy was present for both color and shape targets.

**Table 1:** Linear mixed effects models and results for Experiments 1–3 (significant results in bold).

Experiment	Control variables (Fixed Effects)	Random Effects	Dependent variable	Fixed Effects	Estimate	Likelihood ratio test
Experiment 1	Target type, previous target type, previous accuracy, baseline pupil area, ITI, accuracy (for RT models)	Participant number (intercept), Trial number (intercept)	Accuracy	Mean Dilation	**β = .23, *SE* = .09, *z* = 2.40**	**χ**^**2**^ **(1) = 5.76, *p* = .016**
				Mean Dilation * Target Type	β = -.07, *SE* = .17, *z* = .39	χ^2^ (1) = .15, *p* = .70
			RT	Mean Dilation	**β = -.03, *SE* = .01, *t* = 2.19**	**χ**^**2**^ **(1) = 4.73, *p* = .030**
				Mean Dilation * Target Type	β = .02, *SE* = .02, *t* = .70	χ^2^ (1) = .49, *p* = .48
Experiment 2	Target type, previous target type, previous accuracy, baseline pupil area, ITI, ISI, stimulus duration, accuracy (for RT models)	Participant number (intercept), Trial number (intercept)	Accuracy	Mean Dilation	β = -.01, *SE* = .07, *z* = .17	χ^2^ (1) = .03, *p* = .87
				Mean Dilation * Target Type	β = -.08, *SE* = .12, *z* = .65	χ^2^ (1) = .43, *p* = .51
			RT	Mean Dilation	β = -.01, *SE* = .01, *t* = 1.67	χ^2^ (1) = 2.78, *p* = .10
				Mean Dilation * Target Type	β = -.01, *SE* = .02, *t* = .58	χ^2^ (1) = .34, *p* = .56
Experiment 3	Target type, previous target type, previous accuracy, baseline pupil area, ITI, ISI, color difference, accuracy (for RT models)	Participant number (intercept), Trial number (intercept)	Accuracy	Mean Dilation	β = .02, *SE* = .06, *z* = .42	χ^2^ (1) = .18, *p* = .68
				Mean Dilation * Target Type	β = .08, *SE* = .12, *z* = .66	χ^2^ (1) = .44, *p* = .51
			RT	Mean Dilation	β < .01, *SE* = .01, *t* = .14	χ^2^ (1) = .02, *p* = .89
				Mean Dilation * Target Type	β = .01, *SE* = .01, *t* = 1.08	χ^2^ (1) = 1.17, *p* = .28

Larger pupil dilations also predicted significantly faster response times (β = -.03, *SE* = .01, χ^2^ (1) = 4.73, *p* = .030; see Fig 3D). As with accuracy there was no significant interaction with target type (*p* = .48). There was, however, a significant interaction between mean dilation and accuracy (β = -.08, *SE* = .03, *t* = 2.61, χ^2^ (1) = 6.80, *p* = .009), indicating that, as we might expect, the relationship between mean dilation and RT was stronger for correct trials than incorrect trials.

To summarize, the results of Experiment 1 showed that preparatory pupil dilation is sensitive to the upcoming attention task. Preparing for a difficult shape search task was associated with larger pupil dilations than preparing for an easy color search task. Moreover, pupil dilation was directly linked to performance, as demonstrated by the finding that the magnitude of the dilation predicted both accuracy and response time on a trial-by-trial basis. This latter finding provides initial support for the view that the pupil is not only responding to expectations about upcoming task demands, but is also reflecting online variation in task-specific preparation.

## Experiment 2

In Experiment 1, we found significantly larger pupil dilations for the more difficult shape target relative to the easier color target. We initially interpreted this result to reflect evaluative processing, in which a shape target cue evokes a greater dilation than a color target cue, due to differences in anticipated task difficulty. However, it is also possible that the pupil responds differently to preparation for shape targets than color targets, regardless of anticipated task performance. To establish that a main effect of target type does reflect an evaluative process that is sensitive to expected task difficulty, we took two approaches in two further experiments. In proceeding, we separately tackled the two primary findings of Experiment 1: the main effect of target type on dilation, and the trial-by-trial link between dilation and performance measures (i.e., RT and accuracy). In Experiment 2, we implemented a staircasing procedure that titrated the duration of the search display to produce equivalent performance in the shape and color tasks, with the aim of eliminating differences in expected difficulty. If pupil dilation differed across target type in Experiment 1 because of differences in anticipated task difficulty, then we should no longer see greater dilation for shape than color trials in Experiment 2.

With respect to the relationship between dilation and task performance, we expected that task-specific processing should continue to be present when difficulty is equated across color and shape trials in Experiment 2. Since the trial types were mixed within blocks, participants would be required to implement task-specific preparation to ensure proper configuration on each trial. Therefore, we should continue to see a relationship between dilation and performance outcomes on both shape and color trials (in accuracy and/or RT).

Following from the exploratory findings of Experiment 1, Experiment 2 was run with greater statistical power and with pre-registration. We also moved from using a saccadic response (i.e. fixate on the target) to using a manual two-alternative forced-choice response. This was partly because titrating the search display duration to equate difficulty would sometimes require the stimulus to be removed before the observer had time to make an eye movement. Also, we were concerned that the fixation response would be less reliable than a manual response, and may interfere with the staircasing procedure. Since both saccades and manual RTs both constitute motor responses that are executed following the process of attentional selection, we reasoned that effects of feature-based attentional control should manifest in manual response times in the same way they did for the saccadic responses.

### Method

#### Pre-registration

The experiment methods and analyses were pre-registered prior to data collection on the Open Science Framework website (https://osf.io/m4jqz/, see also S1 File for pre-registered documents).

#### Participants

Thirty-two participants (16 women and 11 men; age range 18–35, *M* = 21.81) were recruited at The Ohio State University, and received $10 in return for participating. The sample size was determined in advance, based on power calculations conducted using G*power [49] with a pilot sample of ten participants. This indicated that 30 participants would be required to detect a significant relationship between pupil dilation and accuracy in the linear effects mixed models with 90% power. To ensure full counterbalancing, the sample size was set at 32 participants. An additional four participants completed the experiment but were excluded and replaced due to a large amount of missing pupil area data (see Experiment 1 Data Analyses for exclusionary criteria).

#### Stimuli and equipment

**Fig 4:**
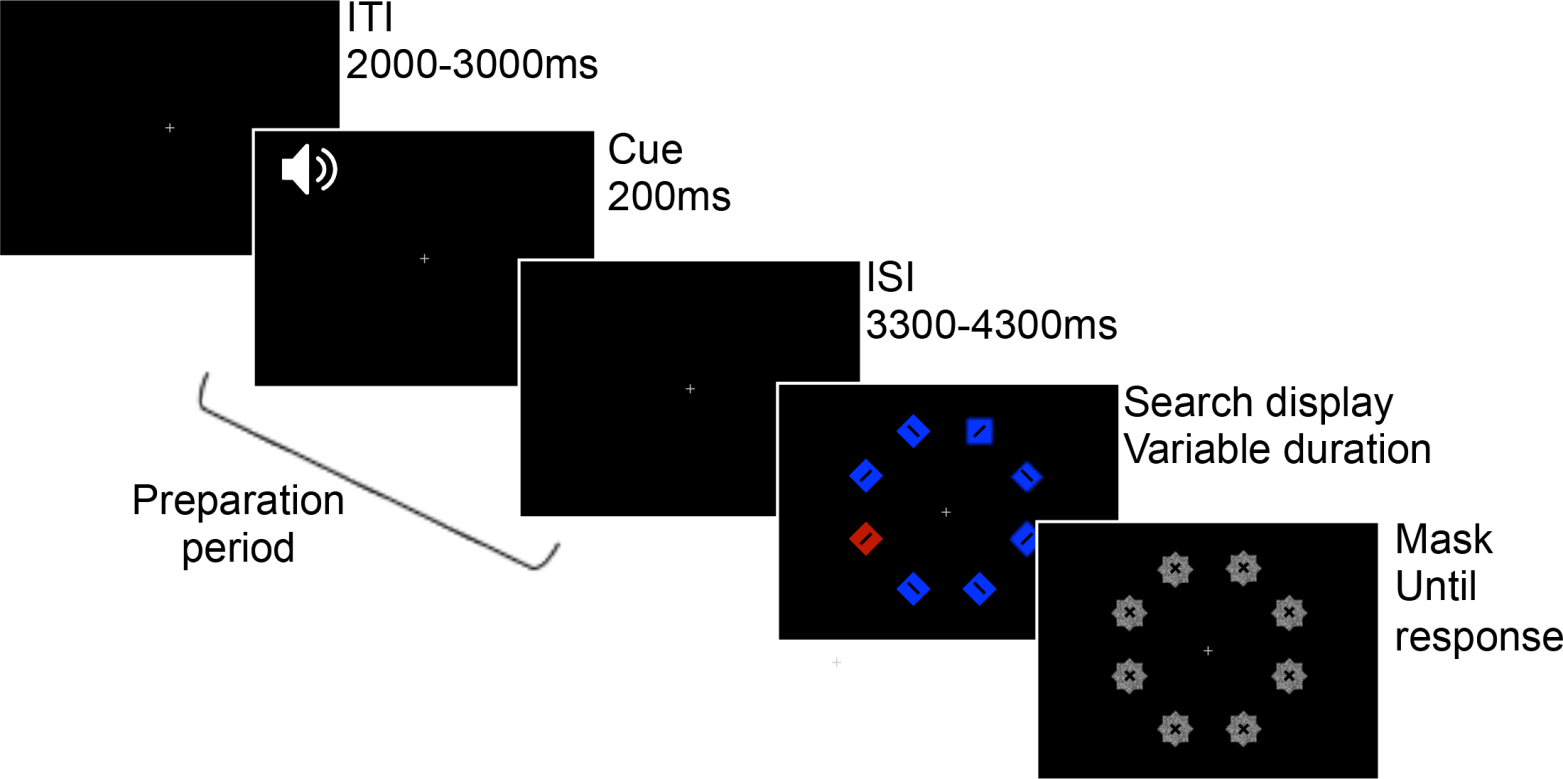
Experiment 2 trial sequence.

The stimuli and equipment were the same as in Experiment 1, with the following exceptions. The search display set size was increased from 6 to 8 items. The singleton values were now counterbalanced across subjects (for shape, square amongst diamonds for half of the participants, and diamond amongst squares for the remaining participants; for color, red amongst blue or blue amongst red). Responses were made by making a two-alternative forced choice key press. Each item in the search display now contained a black bar (0.75° long), oriented 45° to the left or right, and participants judged the orientation of the bar inside the target (Fig 4). A masking display followed the search display and was composed of random-dot noise patches at each of the six locations. The patches were star-shaped (overlapping diamond and square), size 1.9° x 1.9°, and contained both a left and right oriented bar. Participants were free to make eye movements if they wished, but were not instructed to specifically fixate the target.

#### Procedure

The trial sequence was similar to Experiment 1 (see Fig 4). The fixation frame was presented for a variable duration of 2000 or 3000ms, followed by the onset of the auditory cue and preparation period. To encourage early preparation, we introduced variability to the preparation period, which could be 3000, 3500 or 4000ms selected at random. The search display was then presented, and participants were asked to indicate the orientation of the bar inside the target by pressing the N or M keys on the keyboard. Participants were instructed to prioritize accuracy, but to respond quickly once they determined their response. The duration of the search display was varied using the PEST staircasing method [50]. The initial stimulus duration was 588ms for both color and shape trials, and the lower and upper duration bounds were set at 47ms and 1882ms, respectively. Staircasing was performed on color and shape trials independently to converge at accuracy levels of 75% for both targets, using the parameters *W* = 1, maximum step size = 200ms, minimum step size = 12ms, and starting step size = 106ms (see [50] for parameter details). The mask display was presented immediately after the search display until a response was made. Participants were free to respond after the search display was removed. Participants completed 10 blocks of 24 trials, with equal color and shape target trials, and repeat and switch trials, in each block.

#### Analysis of pupil data

The analyses were as per Experiment 1, with the following changes. First, in order to give the staircase procedure ample time to converge at 75%, the first three blocks were excluded from analyses, giving a total of 168 trials. Second, because the preparation period varied between 3000 and 4000ms across trials, pupil dilation was calculated using only the first 3000ms. We also added preparation period into the mixed effects models as a control predictor. Additionally, because the stimulus duration on a given trial was also expected to influence accuracy and RT, this variable was included as another control predictor in the models.

### Results

#### Behavior

For five participants, the staircase could not converge at the intended accuracy level for the shape trials, within our defined stimulus duration boundary, suggesting that the task was too difficult for them (for these participants, the mean stimulus duration for difficult trials was 1880 ms and mean accuracy was 56%). These participants were removed from further analysis, leaving 27 participants. RT analyses were conducted on accurate trials only, excluding RTs < 300ms (.04% of trials) or more than three standard deviations above the mean for color or shape trials (2.76% of trials).

Analysis of accuracy data confirmed that the staircasing procedure yielded 75.49% on easy trials and 75.97% on difficult trials, *t*(26) = .37, *p* = .72, *d* = .07. Shape trials required a significantly longer stimulus duration than color trials (922ms vs 154ms, *t*(26) = 13.41, *p* < .001, *d* = 4.13) and also produced longer response times (1271ms vs 791ms, *t*(26) = 14.63, *p* < .001, *d* = 3.07).

#### Main effect of target type on dilation

**Fig 5:**
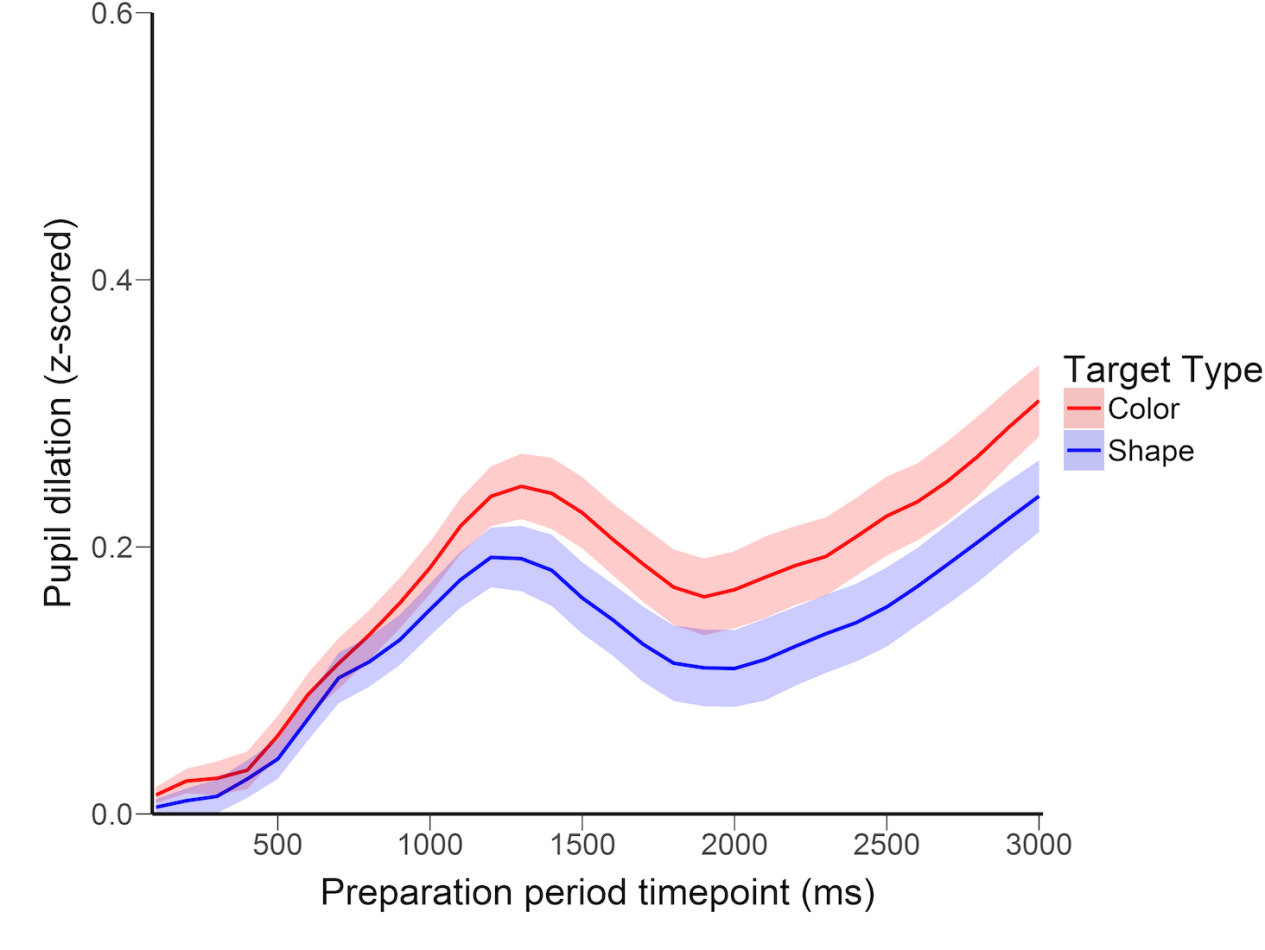
Pupil dilation across the preparation period in Experiment 2. Error bands depict standard error of the mean color vs shape difference scores.

With accuracy equated, difficult trials no longer produced larger pupil dilations. In fact, dilations were significantly larger for color trials, *t*(26) = 2.26, *p* = .032, *d* = .44. As showed in Fig 5, this effect began to emerge relatively early (between 500 and 1000ms after cue onset) and was maintained for the entirety of the analyzed period.

#### Relationship between dilation and task performance

Next we examined whether pupil dilation predicted trial performance. Unlike in Experiment 1, pupil dilation no longer predicted accuracy (*p* = .87; see Fig 3B for accuracy residuals plotted against mean dilation), and there was no interaction between dilation and target type in predicting accuracy (*p* = .51). Similarly, there was no significant relationship between dilation and RT (*p* = .10, see Fig 3E), nor did mean dilation interact with target type (*p* = .56) or accuracy (*p* = .31) in predicting RT.

The failure to find a trial-by-trial relationship between dilation and performance–as we did in Experiment 1 –led us to explore alternative accounts for the findings. One possible explanation is that the mean dilation measure that we used was not sufficiently sensitive. We chose to average dilation across the entire preparatory period largely because we did not want to make assumptions about where in the preparatory period effects would be strongest. But pupil responses are slow to emerge, and inspection of Fig 5 shows that differences in preparatory activity are not apparent until approximately 1500ms into the trial. To examine whether pupil dilation in the later phase of the preparatory period is a more powerful predictor, we conducted a supplementary analysis (i.e., not planned in our pre-registration), using dilation averaged across the second half of the preparatory period only (1500ms to 3000ms). Nevertheless, the pattern of results changed little: there was no relationship between pupil dilation and accuracy (*p* = .65) or interaction between dilation and target type in predicting accuracy (*p* = .37). Pupil dilation did not predict RT (*p* = .10), or interact with target type (*p* = .44) or accuracy (*p* = .48) in RT analyses.

Next, we took a closer look at the methodological differences between Experiments 1 and 2. While Experiment 1 had participants respond by making a saccade to the target, Experiment 2 used a manual response to an orientation judgment. While we had not anticipated it, the added component of making an orientation judgment after locating the target may have altered, or added noise to, the response time distribution. One way to address this is to analyze the Experiment 2 results in the same manner as Experiment 1. That is, in another supplementary analysis, we marked a trial as correct if the first fixation went to the target item and incorrect if it went to a distractor item, and we used saccadic latency in the place of RT. As a disclaimer to presenting this analysis, we acknowledge that this approach is not without limitations. First, given that participants were not required to make a fixation at all in Experiment 2, only a subset of trials were included in the analysis (i.e., those in which a fixation was made, approximately 62%). Second, because the stimulus duration was very short for Color trials, almost all of the saccades made to the target location arrived after the search display had been replaced by the mask. Nevertheless, we reanalyzed the Experiment 2 results using this approach. Again, pupil dilation neither predicted saccadic accuracy (*p* = .27), nor did it interact with target type (*p* = .87). However, larger pupil dilations did predict faster saccadic latencies (β = -.04, *SE* = .02, χ^2^ (1) = 5.41, *p* = .020). This did not interact significantly with accuracy (*p* = .51), but the interaction with target type was marginally significant (*p* = .06). Analyzing color and shape trials separately revealed that pupil dilation predicted saccadic latency for color trials (β = -.09, *SE* = .03, χ^2^ (1) = 8.73, *p* = .003) but not for shape trials (*p* = .42).

Overall, the Experiment 2 results showed that, when the difficulty of the color and shape tasks was equated, preparing to search for the shape target no longer evoked larger pupil dilations. In fact, pupil dilations were larger for color trials than shape trials. While we did not expect this result, one possibility is that a kind of temporal anticipation was also contributing to pupil dilation. Stimulus duration was much shorter for color trials than shape trials (154ms vs 922ms). The expectation that there would be only a brief window of time to shift attention to the target may have further enhanced pupil dilation. Or, it may have prompted earlier preparation and allowed the pupil to reach peak dilation more rapidly. Such effects of temporal anticipation may reflect the activity of an evaluative mechanism.

Regarding the relationship between pupil dilation and performance, the results were mixed. Pupil dilation did not predict manual response accuracy or RT, but it did predict saccadic RT, for color trials only. This may suggest that the relationship is dependent on response mode, and manual responses are simply not sensitive enough to show the relationship. However, as mentioned there are a number of challenges with using saccadic responses in the current study, which precludes us from drawing strong conclusions at this stage. We revisit this issue in Experiment 3 and in the General Discussion.

## Experiment 3

In Experiment 3, we again pursue separate questions relating to the main effect of target type and the link between dilation and performance.

**Fig 6:**
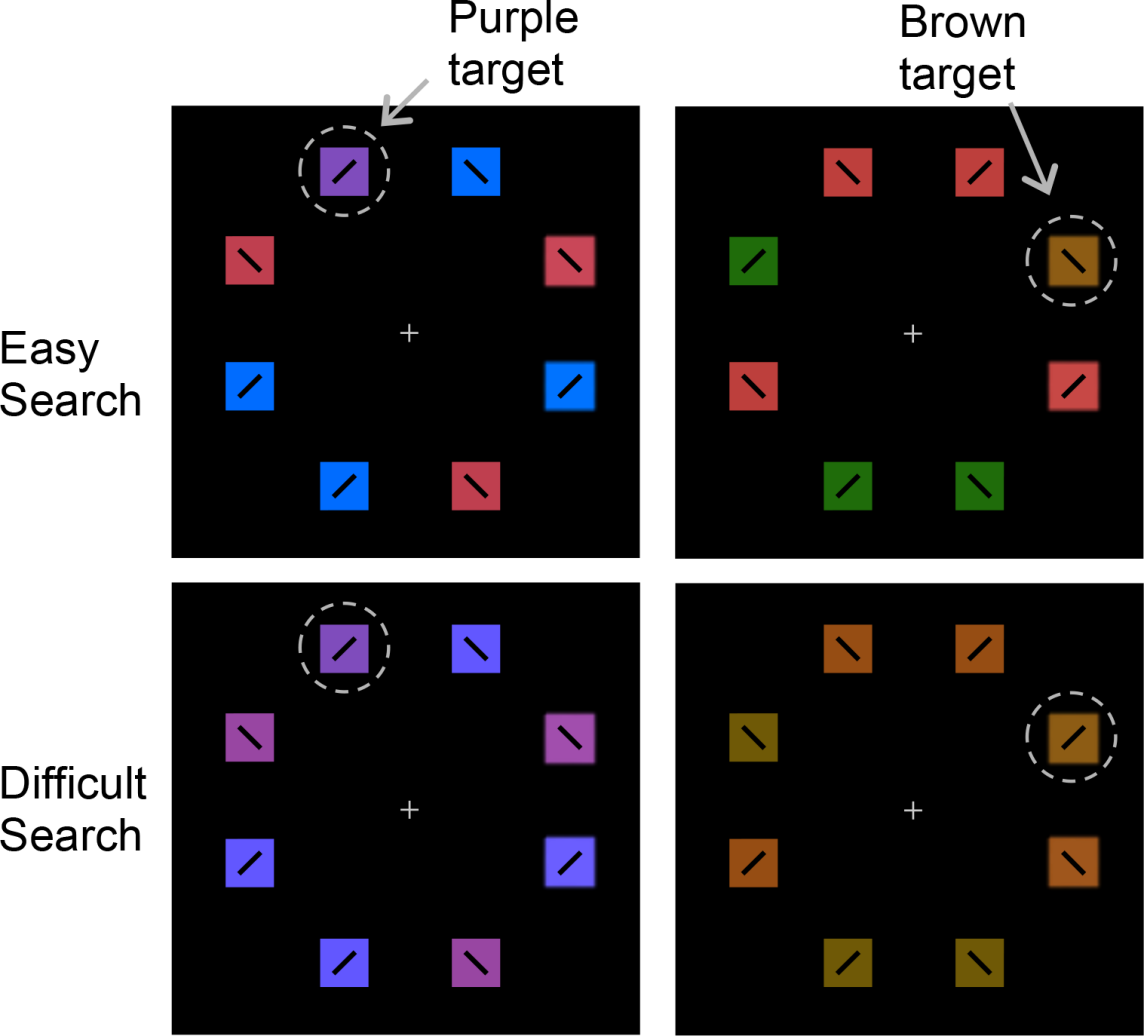
Examples of Experiment 3 stimuli. Target-distractor color similarity was staircased to fix accuracy at 85% in easy search and 65% in difficult search.

First, with respect to the effect of condition, Experiment 2 failed to show greater dilation in advance of shape vs. color targets, when the two were equated for overall task difficulty (in fact, the effect was reversed). This was largely consistent with our interpretation that dilation reflects an evaluative process that responds to anticipated task difficulty. To produce further evidence for this interpretation, in Experiment 3 we used essentially the converse task manipulation as in Experiment 2; specifically, we eliminated categorical differences in the target type (i.e., now two color targets instead of color vs. shape), and we also deliberately manipulated task difficulty across the two conditions. The targets were both defined by a color–one brown and one purple–and were presented amongst non-target colored distractors (Fig 6). To manipulate difficulty, the distractors were made to be more or less similar to the targets. Target-distractor color differences were staircased to fix accuracy at 65% for one target (difficult trial) and 85% for the other target (easy trial). By staircasing color similarity instead of stimulus duration, we avoided the potential effects of large differences in duration on performance encountered in Experiment 2. If expectations of difficulty (evaluative processing) influence pupil dilation, we should again see larger dilations for the difficult target.

Second, we once again tested the relationship between trial-by-trial pupil dilation and task performance. If pupil dilation is sensitive to task-specific preparation, we should see a significant relationship between pupil dilation and search performance.

### Method

#### Pre-registration

The experiment methodology and analyses were pre-registered prior to data collection with the Open Science Framework (https://osf.io/9wh9a/, and see S2 File).

#### Participants

Thirty-two participants (26 female, 6 male; age range = 18–31, *M* = 20.96) completed the experiment in return for $10 compensation. Four additional participants were excluded and replaced due to missing data.

#### Stimuli and equipment

All items in the search display were squares containing black bars oriented 45° to the left or right. Only one target was presented on each trial. Of the seven distractors, four were presented in one distractor color and three in the other distractor color. The target and distractor colors were selected from around a circle in CIE-Lab color space (L = 60, center a = -50, center b = -10, radius = 60). The targets colors were purple (a = 36.96, b = -40.00) and brown (a = 5.52, b = 46.38), spaced 100° apart the circle. At the start of the experiment, the distractors that accompanied each target were the colors 45° clockwise and 45° counterclockwise of the target (for the purple target, distractors were pink (a = 42.96, b = 5.53) and blue (a = 0.53, b = -67.96); for the brown target, distractors were orange-pink (a = 39.38, b = 15.36) and green (a = -40.36, b = 44.38). The angular distance from the distractor colors to the target color was staircased across the experiment using the PEST procedure, with the maximum allowable difference set at 60° and the minimum allowable distance at 1°. The target colors remained constant throughout the experiment, and only the distractor colors were varied. The staircasing was conducted independently for the two targets to achieve 85% accuracy for the easy target and 65% for the difficult target, using the parameters W = 1, maximum step size = 25°, minimum step size = 5°, starting step size = 5°. Stimuli were presented on a 23-inch Acer LCD monitor at a viewing distance of 64cm.

#### Procedure

Participants were informed that they should search for either a purple or a brown target in response to the auditory tone. They were instructed that the color of the distractors would change, but they were not told that there would be any difference in difficulty across two target colors. The assignment of target color to difficulty level and auditory cue was counterbalanced across participants. The trial sequence and response was identical to Experiment 2, with the exception that the search display always remained onscreen for a fixed amount of time before the mask (500ms).

#### Analysis of pupil data

As with Experiment 2, the first three blocks were excluded from analyses to allow the staircasing procedure to converge. All analyses were identical to Experiment 2, except that the control predictor stimulus duration used in the mixed effects models in Experiment 2 was replaced with target-distractor color difference (in degrees).

### Results and discussion

#### Behavior

Analysis of the accuracy data confirmed that the staircasing procedure modulated accuracy as expected: accuracy for the easy target was 85.53%, significantly high than for the difficult target (65.74%, *t*(31) = 29.02, *p* < .001, *d* = 5.13). For RT analyses, incorrect trials and those with RTs > 300ms (.13%) or more than three SD above the mean for easy and difficult trials (2.23%) were removed. Correct trial RT was also faster for easy than difficult targets (875ms vs 1038ms, *t*(31) = 8.42, *p* < .001, *d* = 1.56), and target-distractor color difference was larger on easy trials (22.10° vs 10.03°, *t*(31) = 7.86, p < .001, *d* = 1.80).

#### Main effect of target type on dilation

**Fig 7:**
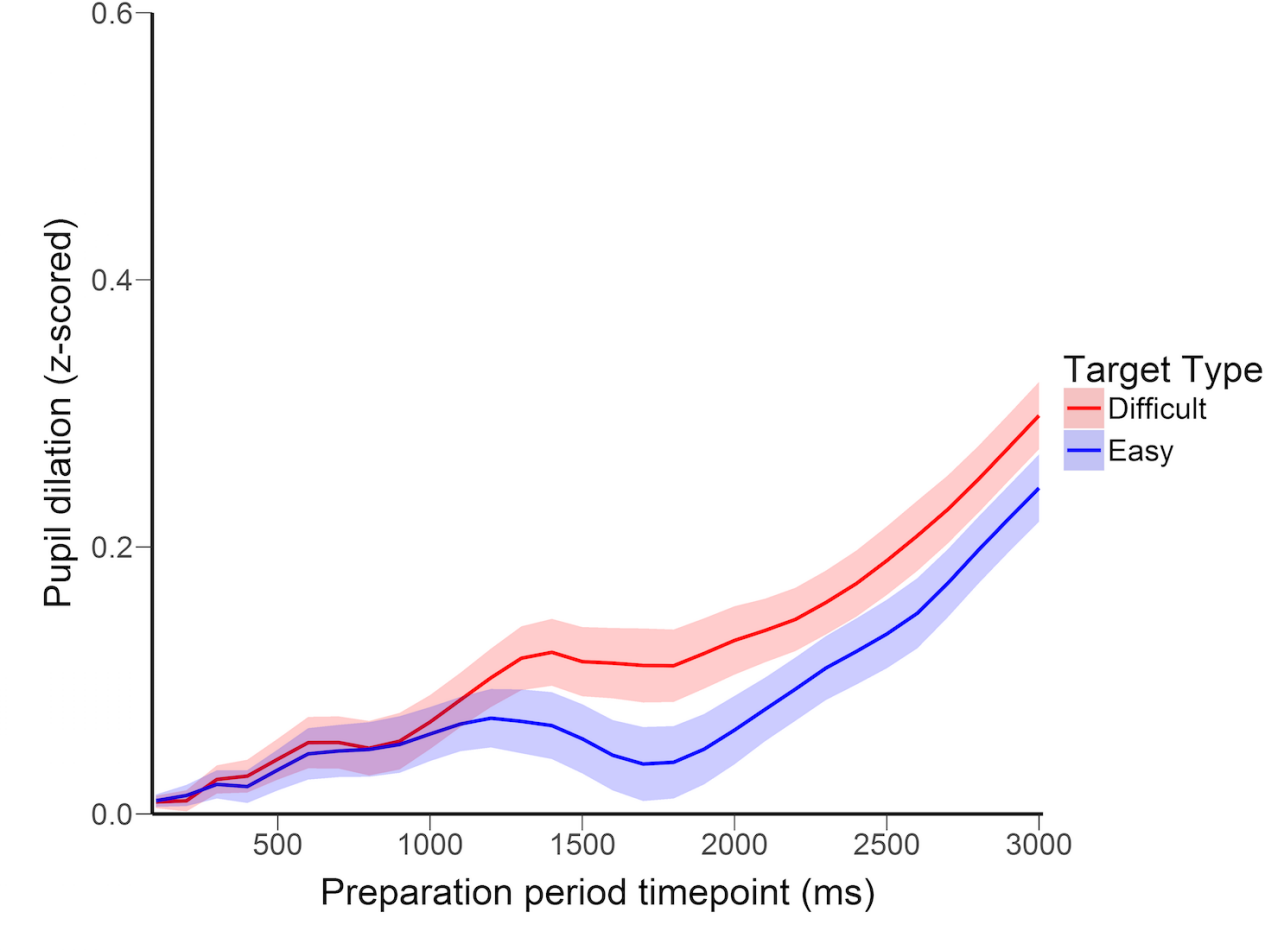
Pupil dilation across the preparation period in Experiment 3. Error bands depict standard error of the mean color vs shape difference scores.

As predicted, target difficulty significantly modulated preparatory pupil dilation. Mean dilation was larger on difficult than easy trials, *t*(31) = 2.24, *p* = .033, *d* = .041, the difference emerging approximately 1000ms after cue onset and remaining steady across the preparation period (Fig 7).

#### Relationship between dilation and task performance

As with Experiment 2, we found little evidence that preparatory pupil dilation predicted trial-by-trial performance. For accuracy, the fixed effect of pupil dilation was not significant (*p* = .67, see Fig 3C), nor was there an interaction with target type (*p* = .51). This was also the case for RT (fixed effect *p* = .88, interaction with target type *p* = .28; interaction with accuracy *p* = .44; see Fig 3F).

As we did in Experiment 2, here we again carried out two supplementary analyses that were not included in the pre-registration. First, we reran the analyses using mean dilation from the second half of the preparatory period (1500-3000ms) only. We found no relationship between pupil dilation and accuracy (*p* = .57) and no interaction with target type in predicting accuracy (*p* = .36). For RT analyses, mean dilation was not a significant predictor (*p* = .95), nor did it interact with target type (*p* = .26) or accuracy (*p* = .38). In the second post hoc analysis, we analyzed the relationship between pupil dilation and saccadic accuracy and latency. We included only trials in which an item was fixated (65% of trials). Consistent with the manual RT data, we found no relationship with performance. Pupil dilation was not related to saccadic accuracy (*p* = .33), nor did it interact with target type (*p* = .26). Similarly, it did not predict saccadic latency (*p* = .74) or interact with accuracy (*p* = .75). The interaction with target type in predicting RT approached significance (β = -.02, *SE* = .01, χ^2^ (1) = 3.00, *p* = .083), however simple effects analyses conducted separately at each target type showed that pupil dilation was not a significant predictor for either Easy (*p* = .69) or Difficult (*p* = .91) trials alone.

## General discussion

Pupil dilation has long been used as an index of task demands, during both task execution (e.g. [25–27]) and task preparation (e.g. [38, 39]). Here we asked whether pupil dilation can provide insights into attention task preparation. Across three experiments, participants were cued to search for one of two possible targets on each trial, and we measured pupil dilation during a preparation period between cue onset and search display onset. In the first exploratory experiment, the two targets, a color singleton and a shape singleton, varied in their degree of search difficulty. We found that pupil dilation was larger in preparation for the more difficult, low-salience shape singleton. In Experiment 2, we attempted to eliminate the differences in difficulty across the two tasks by independently staircasing the duration of the search display. Under these conditions, low-salience shape targets no longer evoked larger dilations, and in fact the relationship was reversed, confirming that pupil dilation is not solely dependent on target properties. In Experiment 3, we manipulated difficulty within a single feature dimension by adjusting the similarity of the distractors colors to the target. Preparatory pupil dilation was again larger for the difficult search.

Together these results demonstrate that pupil dilation in preparation for an attention task responds to expectations about the difficulty of the search task. We attribute this effect to an evaluative mechanism that assesses the demands of ongoing performance and decides whether task-level control should be adjusted [1–4]. Other studies have shown variation in pupil dilation on processes thought to rely on this mechanism (e.g. conflict processing, [26]; proactive control [51]). The brain region most often implicated in this evaluative process, the ACC, is one of the main sources of input to the locus coeruleus, which in turn plays a critical role in controlling pupil size [22, 52, 53]. Further, recent monkey studies have shown a relationship between ACC activity and variation in pupil size [54, 55]. Our results suggest that this mechanism is active in preparation for visual search, and can be reliably accessed by measuring pupil dilation.

A surprising finding emerged in Experiment 2, in which accuracy was matched for color and shape targets, and thus no difference between conditions would be expected. Instead, color targets produced a larger dilation than shape targets. This finding implies that the evaluative mechanism may be sensitive to other factors in addition to task accuracy. Stimulus duration was very short for the color target displays, and it is possible that the anticipation of having to attend and act very quickly may have increased the expectation of difficulty relative to the shape target. Such a possibility presents an interesting avenue for exploring of evaluative effects in future studies.

An interesting remaining question is the extent to which the difficulty effects were driven by implicit and/or explicit learning. Although we did not tell participants that the two targets would differ in difficulty, we assumed that participants would quickly learn these differences in an explicit manner. It is well established that color singletons are much more salient than shape singletons [40], and most participants in our experiments mentioned anecdotally that they did indeed find the “difficult target” to be more difficult. Thus, the variation in preparatory activity for the two tasks may have been driven by explicit expectations of difficulty. On the other hand, it also possible that explicit awareness of the relative task difficulty is not necessary. That is, implicit knowledge alone could be sufficient to influence preparatory processing. Future experiments could test the relative contributions of implicit and explicit learning by manipulating the two factors independently. For instance, one approach could be to equate performance as we did in Experiment 2 but falsely inform participants that one of the two targets is more difficult that the other.

Overall we found mixed evidence that pupil dilation indexes task-specific preparation for visual search, which in this case entails the configuration of attentional control settings. If pupil dilation were truly linked to task-specific preparation, we would expect it to correlate with search performance. That is, the more preparation performed by the attentional control system, the faster and more accurate the search process. This was the case in Experiment 1, where dilation predicted trial-by-trial accuracy and response time. However, the fact that we did not consistently replicate these results in Experiments 2 and 3, both of which had considerably more power, leads us to question the reliability of the Experiment 1 finding. One possibility is that differences in response mode prevented the relationship from emerging in Experiments 2 and 3. Experiment 1 used a saccadic response, while Experiments 2 and 3 used a manual response. The manual responses required additional processing, including target location, orientation identification and response initiation, which may have added noise and concealed the relationship with performance. Saccadic-based responding, on the other hand, has been linked to preparatory pupil dilation in two other studies [37, 38], and a direct link has been postulated between brain areas involved in oculomotor preparation (frontal eye fields and superior colliculus) and pupil dilation control [38]. Consistent with this hypothesis, when Experiments 2 was analyzed using saccadic responses, we found a significant relationship between pupil dilation and saccadic RT for color targets. However, in Experiment 3, in which both targets were also defined by a unique color, this relationship was no longer present. Moreover, there was no relationship with saccadic accuracy in either experiment, and so we cannot draw any firm conclusions about whether pupil dilation is indexing task-specific preparation. We also note that if the relationship between pupil dilation and performance hinges on response mode, then pupil dilation may not be the most reliable measure for accessing task-specific preparation in future studies, especially when a correlation between task-specific preparation and performance has been demonstrated using manual responses in other domains (e.g. fMRI [10, 11, 14, 41]).

While we used pupil dilation to make inferences about task demands and the engagement of cognitive resources or effort, we cannot rule out the possibility that it reflects other processes. Pupil dilation has been related to a variety of other cognitive functions, including physiological arousal [56], emotional responding [57] and responding to rewarding stimuli [58]. Teasing apart these distinct functions is not trivial, and may sometimes be causally related; a high degree of effort may necessitate high arousal, for example. Future work will be required to assess these alternative accounts.

In summary, we show that pupil dilation is modulated by expected difficulty in a visual search task, suggesting that it is sensitive to evaluative mechanisms of control. The findings highlight the utility and reliability of pupil measurements in the study of preparatory processing.

## Supporting information

S1 File Experiment 2 pre-registration documents. (PDF)

S2 File Experiment 3 pre-registration documents. (PDF)
