# Supplementary material for: Pre-stimulus pupil dilation and the preparatory control of attention
Source: PLoS One. 2017 Dec 8;12(12):e0188787. doi: 10.1371/journal.pone.0188787 (PMC5722334; doi:10.1371/journal.pone.0188787)
Supplement: S1 File — (PDF) [file pone.0188787.s001.pdf]

Supporting Information 1: Experiment 2 Pre-registration documents

Pre-registered 7/26/2016 on the Open Science Framework
https://osf.io/m4jqz/

1. Summary

Study Information

1. **Title**

Pupil dilation in preparation for visual search

2. **Authorship**

Jessica Irons & Andrew Leber

3. **Research Questions**

See Rationale and Hypotheses [below]

3.1 Does preparing attentional control mechanisms for a difficult visual search (low salience target) require more effort than preparing for an easier search (high salience target)?

3.2 Does effort expenditure during preparation for visual search predict search performance (accuracy and response time)?

4. **Hypotheses**

4.1 Preparing to search for a difficult target (low salience shape target) will be associated with a larger pupil size than preparing for an easy target (high salience color target).

4.2 Larger pupil dilations will predict higher accuracy/faster RT, especially for difficult targets.

Sampling Plan

5. **Existing data**

5.1.1. Registration prior to creation of data: As of the date of submission of this research plan for preregistration, the data have not yet been collected, created, or realized.

6. **Data collection procedures.**

6.1.

6.1.1. *Timeline.* The study will take place during summer and fall 2016.

6.1.2. *Participants.* Participants will be recruited through the Ohio State University first-year participant scheme and via flyers posted around campus. Participants will be aged between 18-40, have normal or corrected-to-normal vision and normal color vision.

7. **Sample size**

7.1. Thirty-two participants.

8. **Sample size rationale**

8.1. Sample size is based on a power analysis conducted using data from a pilot experiment with 10 participants. Approximately 30 participants are needed to provide 90% power of detecting a significant relationship between pupil dilation and performance (accuracy and response time) in the linear mixed model proposed below. To allow for full counter-balancing, we will collect data from 32 participants. Data from participants will be excluded if we are unable to properly track, if more than 40% of their eye tracking data is missing (ie due to blinks or calibration failures), if they fail to follow instructions, or if their accuracy on either target type is at floor (ie the staircase cannot converge). Data from any missing participants will be replaced to keep the sample size at 30 participants.

Variables

9. **Manipulated variables**

9.1. Primary independent variable: Target Type: Easy (high salience color target) v Difficult (low salience shape target)

9.2. Secondary independent variable: Intertrial condition: Repeat (same target as previous trial) v Switch (different target from previous trial)

10. **Measured variables**

10.1. Response accuracy

10.2. Response time

10.3. Mean pupil dilation

Design Plan

11. **Study type**

11.1. Experiment

12. **Blinding**

12.1.1. No blinding is involved in this study (repeated measured study, all participants complete all conditions).

13. **Study design**

13.1. Repeated measured design. Two variables, target color (red or blue) and target shape (square or diamond), will be counterbalanced.
13.2. See PS25 Method for detailed method information

Analysis Plan

14. Statistical models

14.1. Hypothesis 1: We will compare mean dilation in the Easy and Hard conditions using a paired-samples t-test.
14.2. Hypothesis 2: To assess whether pupil dilation predicts performance, we will use linear mixed-effects models, with response time and accuracy as two separate dependent variables, and subject number as a random effect predictor. Our main predictors of interest will be mean pupil dilation, and the interaction between mean dilation and target type. First, to reduce noise and control for extraneous variables, we will firstly build a reduced model with a number of control predictors (such as trial number, baseline pupil area etc), and use standard AIC/BIC measures to find the model that best accounts for the variance. Second, we will add mean dilation as a fixed effect, and use a standard likelihood ratio test to assess whether adding mean dilation accounts for significantly more variance than the reduced model. We also predict that the effect of mean dilation will be stronger for Hard targets than Easy targets. To test this we will firstly assess whether the effect of mean dilation depends on target type (easy v hard), by adding a mean dilation*target type interaction term and comparing this to the model without the interaction term using a likelihood ratio test. Next, we will then test the effect of mean dilation separately for easy trials only and hard trials only.

15. Transformations

15.1. Pupil dilation will be z-scored according to the procedure in the PS25 analysis plan. For the linear effects models, categorical variables will be dummy-coded. Some control predictors (such as trial number) may be rescaled and centered to reduce scale variation across the predictors.

16. Follow-up analyses

16.1. To test whether the previous target type affects pupil dilation on the current trial, we will compare pupil dilation across Switch and Repeat trials using a 2 (target type) x 2 (intertrial conditions: switch and repeat) within subjects ANOVA. If the interaction is significant, paired comparison comparing switch and repeat will be conducted at each level of target type.

17. Inference criteria

17.1. All tests will be t-tailed and compared against a p-value of .05. The Holm-Bonferroni method will be used for correcting for multiple-comparisons.

18. Data exclusion

18.1. Participants with >40% pupil data missing (e.g. due to blinks) will be excluded

18.2. For RT analyses, only correct trials will be analyzed.

2. Rationale & Hypotheses

Attentional control settings bias the visual system to prioritize task-relevant features and properties in the environment. While a great deal of research has investigated the kinds of control settings at our disposal (e.g., shape, color, oddballs, etc), little is known about how individuals choose these settings. Some researchers (e.g. Bacon & Egeth, 1994) have speculated that we choose the settings requiring the least cognitive effort. However, before this speculation can be addressed, it is necessary to determine if the amount of effort required to establish control varies across different control settings.

Here, we aim to use pupillometry to measure preparatory effort for two types of control settings. Pupil dilation has been used in many studies as a marker of cognitive effort (e.g. Kahneman, 1973). Previous experiments in our lab suggest that preparing for a difficult search is associated with larger pupil dilation than preparing for an easy search. Here we extend this to also examine whether pupil dilation predicts task performance on a trial-by-trial basis.

Hypothesis 1: Preparing to search for a difficult target (low salience shape target) will be associated with a larger pupil size than preparing for an easy target (high salience color target).

Hypothesis 2: Larger pupil dilations will predict higher accuracy/faster RT, especially for difficult targets.

3. Methods

Participants

- Data from a pilot experiment with 10 participants indicate that approximately 30 participants are needed to provide sufficient power (90%) to detect a significant relationship between pupil dilation and accuracy in the

linear mixed model. To ensure full counterbalancing, we will run 32 participants.

Stimuli

- Visual search display composed of eight items in ring around fixation
- Two targets present on every trial
  - **Easy color singleton**: red amongst blue or blue amongst red (counterbalanced across participants)
  - **Difficult shape singleton**: square amongst diamonds or diamonds amongst squares (counterbalanced across participants)
- Black line appears in each item, oriented to left (\) or right (/).
- A high pitch and low pitch tone are played at the beginning of each trial to signal the target. Mapping of tone to target types is counterbalanced across participants.

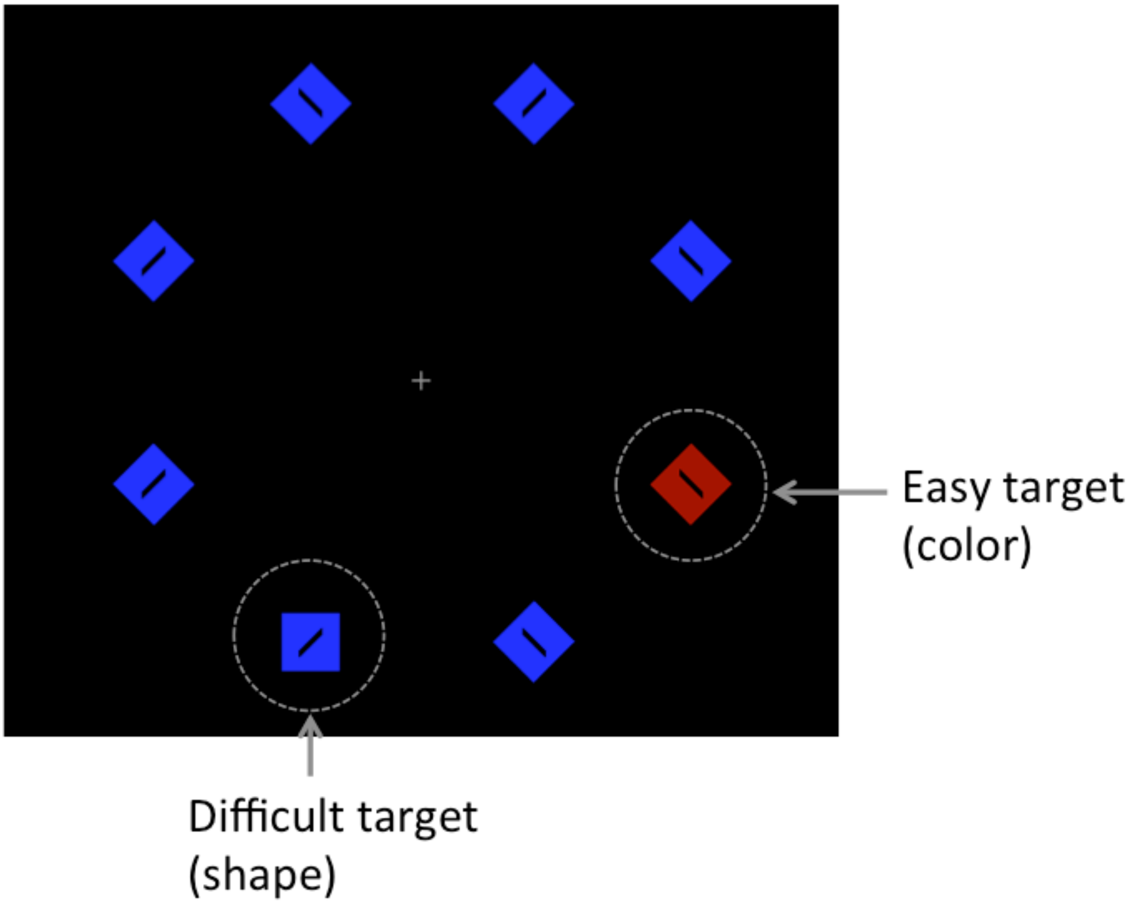

Apparatus

- 20-inch monitor
- Eyelink 1000 eye tracker

Procedure

- Target for each trial is cued by a tone.
- Participants fixate central fixation point during preparation.
- When search display is presented, judge whether the bar inside the target is oriented to the left or right, and respond with keypress.
- Duration of search display is staircased separately for difficult and easy targets to keep accuracy at 75%. Uses PEST procedure for staircasing (see Taylor & Creelman, 1967).
- Within each block, half of the trials are easy and half are hard.

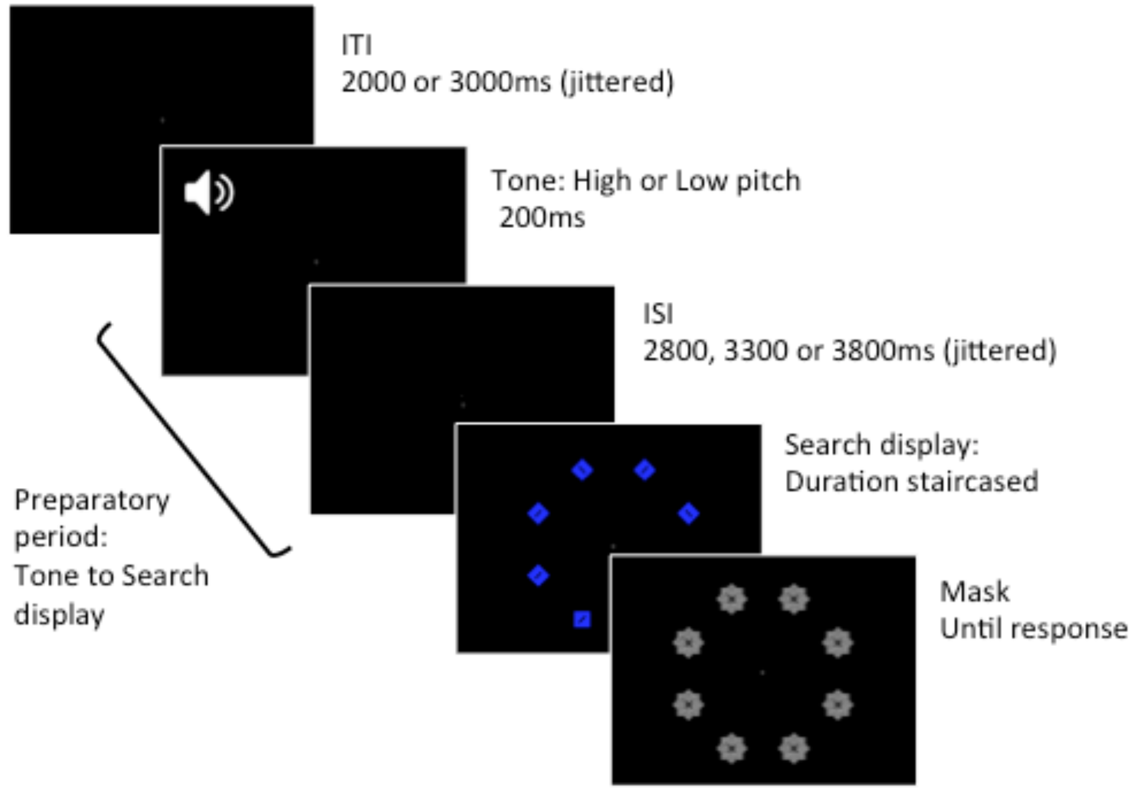

4. Planned Analyses

Pre-processing of pupil data

- Pupil area will be measured every 2ms.
- Any participants with > 40% data missing (e.g. due to blinks) are removed (Nassat, Rumsey, Wilson, Parikh, Heasly & Gold, 2012).

- Blink removal:
    - o Eyelink software use to identify blink start and end period.
    - o An additional 60ms before blink and 150ms after blink is included in blink period (Cavanagh, Wiecki, Kochar & Frank, 2014; Siegle, Ichikawa, & Steinhauer, 2008).
    - o Linear interpolation used to replace blinks.
- Pupil area will be downsampled to 10Hz by taking the median pupil area for every 100ms bin.
- All downsampled pupil medians from 100ms prior to the preparatory period to the end of the preparatory period will be z-scored.
- Dilations will be calculated by subtracting each z-scored pupil area in the preparatory period from baseline (100ms prior to tone onset).
- Finally, for the analyses below, we will determine the mean pupil dilation across the preparatory period for every trial.

Primary analyses

- Comparing preparatory pupil dilation across trial types
    - o Mean pupil dilation for easy and difficult targets will be averaged, and compared in a within-subjects t-test (two-tailed, using $p < .05$ for significance).
- Using dilation to predict performance on a trial-by-trial basis
    - o We will use linear mixed-effects models, with response time and accuracy as two separate dependent variables, and by-subject as a random effect predictor.
    - o Our main predictors of interest will be mean dilation, and the interaction between mean dilation and target type.
    - o First, to reduce noise and control for extraneous variables, we will firstly build a reduced model with a number of control predictors (such as trial number, baseline pupil area etc), and use standard AIC/BIC measures to find the model that best accounts for the variance.
    - o Second, we will add mean dilation as a fixed effect, and use a standard likelihood ratio test to assess whether adding mean dilation accounts for significantly more variance than the reduced model.
    - o We also predict that the effect of mean dilation will be stronger for Hard targets than Easy targets. To test this we will firstly assess whether the effect of mean dilation depends on target type (easy v hard), by adding a mean dilation*target type interaction term and comparing this to the model without the interaction term using a likelihood ratio test.
    - o Next, we will then test the effect of mean dilation separately for easy trials only and hard trials only.

Secondary analyses

- Comparing switch and repeat trials

- The size of the pupil dilation may vary depending on whether the trial is a repeat trial (same target as the previous trial) or switch trial (different target to the previous trial).
- This will be assessed using a 2 (target type: Easy vs Difficult) x 2 (across-trial condition: Switch vs Repeat) within-subjects ANOVA with mean dilation as DV (two-tailed, using $p < .05$ for significance). If the interaction is significant, follow-up comparisons comparing switch and repeat will be conducted within each target type (using Holm-Bonferroni correction to keep family-wise error rate at .05).
